# Spatiotemporal analysis of African swine fever in wild boar in Serbia from 2020 to 2024

**DOI:** 10.4102/ojvr.v92i1.2209

**Published:** 2025-02-28

**Authors:** Dimitrije Glišić, Sofija Šolaja, Ljubisa Veljović, Jelena Maksimović-Zorić, Vesna Milićević

**Affiliations:** 1 Department of Virology, Institute of Veterinary Medicine of Serbia, Belgrade, Serbia

**Keywords:** African swine fever, spatiotemporal analysis, epidemiology, surveillance, Serbia

## Abstract

African swine fever (ASF) is a highly fatal viral haemorrhagic disease affecting pigs. This study uses official surveillance data to investigate the persistence and spread of ASF in wild boar populations in Serbia from 2020 to 2024. A total of 480 ASF cases were analysed, with spatiotemporal clustering conducted via SaTScan™ and proximity analyses performed in the Quantum Geographic Information System. The yearly prevalence of ASF in wild boar rose steadily from 0.66% in 2020 to 1.47% in 2023. Seasonal trends showed a significant increase in cases during winter (68%) and spring (24%). Five distinct clusters were identified, predominantly near the borders with North Macedonia and Bulgaria, suggesting potential cross-border transmission. Proximity to major roads was significantly associated with ASF case distribution (*p* < 0.01), while proximity to water bodies and elevation showed no consistent pattern. The findings highlight critical gaps in current passive surveillance systems, which likely underestimate ASF prevalence. The study underscores the need for enhanced surveillance in remote and wooded areas and proposes strategies to improve ASF monitoring and control in wild boar populations.

**Contribution:**

This study highlights the feasibility of cost-effective, non-invasive surveillance methods for ASF detection, offering critical insights for low-income countries where resources for intensive disease monitoring are limited. By demonstrating how environmental and anthropogenic factors drive ASF dynamics, this research provides actionable strategies for improving regional and global ASF control efforts.

## Introduction

African swine fever (ASF) is a viral haemorrhagic disease affecting members of the *Sus scrofa* family, characterised by a high fatality rate. It is one of the most important animal diseases with an incredibly high economic impact on swine production. In China alone, the economic damage was estimated at approximately $111.2 billion during 2018–2019 (You et al. 2021). The disease is caused by the virus of the same name, which is classified within the *Asfarviridae* family and the *Asfivirus* genus (Alonso et al. 2018). The current pandemic is caused by genotype II, which spread outside of Africa in 2007, when it was first detected in Georgia (Rowlands et al. 2008). The virus spread into Russia, infecting wild boar and domestic pigs, where it circulated for the next 4 years, before spreading further into Europe. The first case in Europe was reported in wild boar in 2014 in Lithuania, and since then there have been reports in 20 European countries (World Organisation for Animal Health 2024). The index case of ASF in Serbia was recorded in 2019 affecting domestic pig populations. Swift containment measures were implemented, including culling infected pigs, leading to the prompt resolution of the outbreak (Milićević et al. 2019). The initial detection of ASF in wild boar occurred in January 2020. That year, 43 cases of ASF were reported in wild boar, along with 16 outbreaks in domestic pigs. The following year, 2021, there was an increase in outbreaks in domestic pigs and wild boar cases to 33 and 70, respectively. The upward trend continued into 2022, culminating in 107 outbreaks in domestic pigs and 113 cases in wild boar. The epidemic reached its zenith in 2023 with 991 outbreaks in domestic pigs, including incidents on several large commercial farms, and a peak of 173 cases in wild boar (European Commission: Animal Disease Information System 2019, 2020, 2021, 2022, 2024). According to data from the Statistical Office of Serbia, the country’s wild boar population stands at 39.943 with 20.560 included in the annual hunting quotas. Smallholder farms constitute nearly 40% of all registered farms in Serbia. Biosecurity measures at these farms are often minimal (Glišić et al. 2023b). Pigs are often kept in pens until slaughter, but in some cases, they are kept in pastures with low-security fences that are insufficient to prevent contact with wildlife, potentially leading to interactions between wild boar and domestic pigs. Previous studies of ASF in the country focussed on the transmission of ASF across state borders and primarily anthropogenic factors involved in ASF spread towards domestic pigs (Glišić et al. 2023b; Milićević et al. 2019; Nešković et al. 2021). Seasonality of ASF in wild boar within the European Union (EU) has been previously studied, revealing a notable increase in cases during the winter months in countries such as Poland, Slovakia, Hungary and the Baltic states. However, no distinct seasonal pattern has been observed in Germany and Romania (Ståhl et al. 2023). The distribution of ASF cases in wild boars, including their proximity to waterbodies, forests and roads, has not been previously studied in the country. However, data from the Baltic states suggest that roads and forests may be positive predictive factors for detecting ASF-positive wild boar carcasses (Rogoll et al. 2024). Although the number of ASF cases in wild boar has been steadily increasing, there is no direct evidence linking these cases to the transmission of the virus to domestic pigs. Current research indicates that the primary drivers of ASF spread are irresponsible pig farming practices and anthropogenic factors, such as the illegal transportation of animals (Glišić et al. 2023b). Prior to this research, no studies had been conducted within the country on the spread of ASF among the wild boar population or on the seasonality of ASF infections in these animals. In this study, we aim to assess the prevalence and spatiotemporal spread of ASF in the wild boar population from 2020 to 2024 in Serbia.

## Research methods and design

In this study, we analysed available data regarding outbreaks of ASF in wild boar in Serbia. The number of cases and their coordinates were submitted to the Institute of Veterinary Medicine of Serbia, the National Reference Laboratory for ASF, as part of a national monitoring programme. Cases of ASF infection were detected in Serbia’s regional veterinary institutes by real-time polymerase chain reaction (PCR) method, targeting the p72 gene, with a Ct threshold of ≤ 35 for positive results. The data included cases from January 2020 to August 2024. In total 480 cases of ASF in wild boar have been included in the study. Out of the 480 wild boar, 155 (32.3%) were found dead, while 325 (68.7%) were culled as part of sanitary hunting practices. The estimated number of wild boar per region, as well as the number of square miles per region were obtained from the Statistical Office of Serbia. For each year, 2020–2024, an estimated prevalence was calculated. The number of outbreaks for each month per year estimated the seasonality of ASF. A Chi-square test of independence was performed to evaluate the association between the number of detected cases and seasonal variation. The test compared observed case frequencies across four seasons (winter, spring, summer and autumn) against expected frequencies, with a significance level set at *p* < 0.01.

### SaTScan™

For the spatiotemporal analysis, we utilised SaTScan™ software version 10.1. Prior to running the analysis, we prepared the dataset, accurately geocoding each sample with coordinates and collection dates. Each case was treated as a distinct event. The analysis period was defined from January 2020 to December 2024, with a temporal resolution set to ‘Month’ to capture monthly variations. We conducted a Space-Time analysis using the Space-Time permutation probability model, which is suited for detecting areas with unusually high concentrations of cases over time. To determine statistical significance, we applied the standard Monte Carlo method, conducting 999 replications to infer the *p*-value, only cluster with a *p*-value less than 0.05. The spatial and temporal scanning windows were both set at 50% of the study period, allowing the software to examine potential clusters across half of the total time frame and spatial extent. In addition, to ensure clarity in identifying significant clusters, the hierarchical reporting option was configured to prevent the centres of identified clusters from overlapping with those of other clusters. This approach helps in accurately distinguishing and reporting areas with the most significant increases in case rates.

### Quantum geographic information system

For the estimation of the number of wild boar cases reported near roads and water bodies, official road maps were used in the Quantum Geographic Information System (QGIS), excluding trails from the analysis. Buffers of 1 km and 5 km were created around each road, and the coordinates of positive wild boar cases were overlaid onto these buffers to count the cases within each zone. Similarly, to assess proximity to water bodies, a water layer for the country was downloaded from the GeoFabrik website (https://www.geofabrik.de/). Buffers of 500 m and 1 km were created around each water body (including rivers, riverbeds, reservoirs and lakes), and cases within these buffer zones were counted. To evaluate the impact of proximity to roads on the number of positive cases, a Chi-square test of independence was conducted. The test compared the observed distribution of cases within the buffer zones to an expected distribution, assuming that proximity to roads does not influence case distribution. The null hypothesis posited that the distribution of cases is independent of their proximity to roads. Statistical significance was determined with a *p*-value threshold of 0.01. For water bodies, a similar Chi-square test was conducted to assess whether proximity influenced the distribution of cases, with the null hypothesis assuming an equal distribution of cases regardless of distance to water bodies. Furthermore, the influence of elevation on the number of positive cases was analysed using a raster file of the country’s elevation, which was also obtained from GeoFabrik (https://www.geofabrik.de/). The elevation data were categorised into five zones: zone 0 (< 100 m), zone 1 (100 m – 499 m), zone 2 (500 m – 999 m), zone 3 (1000 m – 1499 m) and zone 4 (≥ 1500 m). The number of cases in each elevation zone was then determined and visually represented using colour coding.

### Ethical considerations

This article followed all ethical standards for research without direct contact with human or animal subjects.

## Results

The annual percentage of ASF-positive wild boar was calculated as 0.66% in 2020, 0.76% in 2021, 1.08% in 2022 and 1.47% in 2023. The percentage of positive samples for 2024 was not calculated because of a disproportionate number of detected cases being confined to a single hunting ground.

**FIGURE 1:**
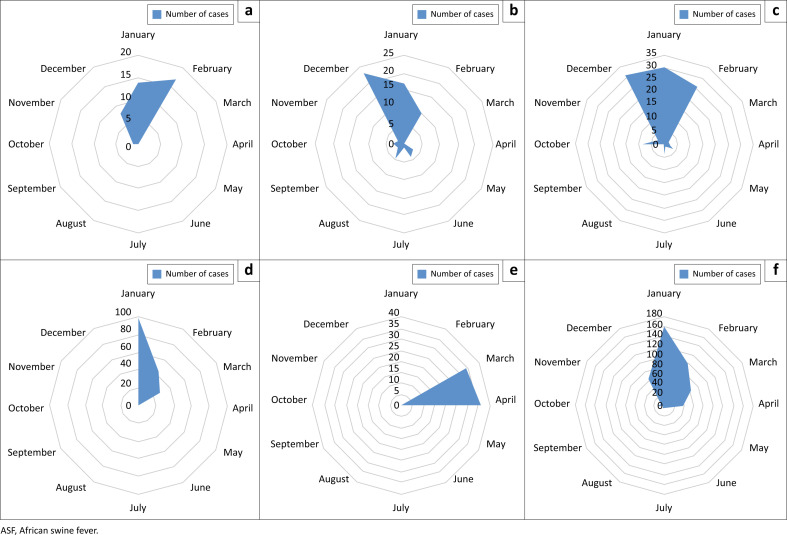
Seasonal Trends. Radar charts illustrating the annual number of ASF cases are shown for each year, providing a clear depiction of seasonal patterns. The final radar chart offers a summary of the overall seasonal trends observed from 2020 to 2024, highlighting recurring patterns over the study period. (a) year 2020, (b) year 2021, (c) year 2022, (d) year 2023, (e) year 2024, (f) combined trends from 2020–2024.

**FIGURE 2:**
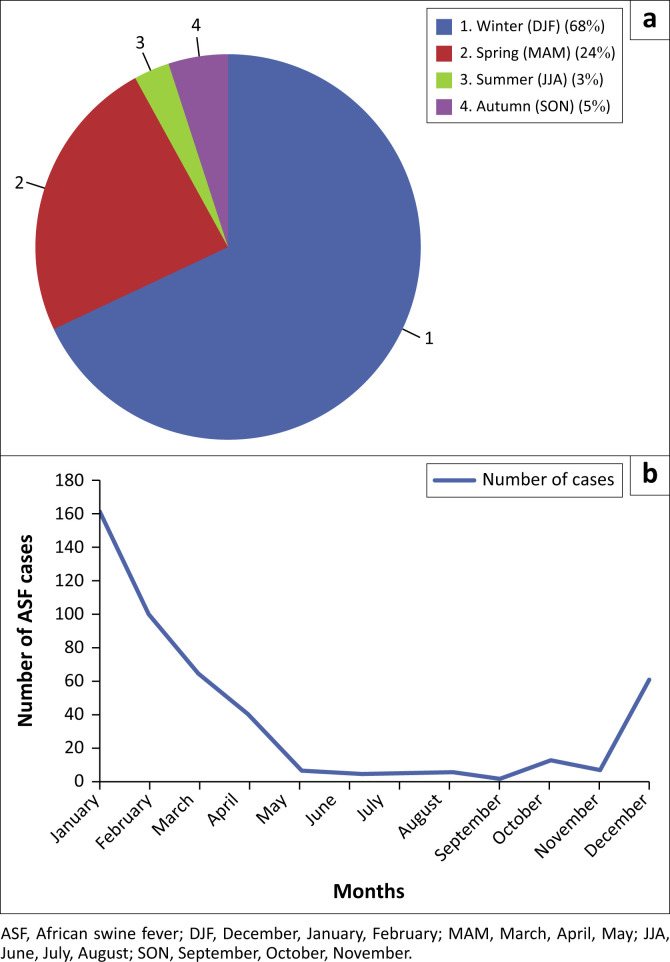
(a) A pie chart showing the proportion of ASF cases recorded in each season: winter (December–February), spring (March-May), summer (June–August), and autumn (September–November). (b) A line graph illustrating the total number of ASF cases from 2020 to 2024, highlighting the temporal distribution of cases over this period.

A Chi-square test was conducted to assess the association between case distribution and seasons, revealing a highly significant relationship (Chi^2^ = 519.324, *p* < 0.00001), suggesting a strong deviation from the expected seasonal distribution. The majority of cases (68%, or 321 cases) were detected during the winter months of December, January and February. This was followed by the spring months, which accounted for 24% (110 cases), autumn with 5% (22 cases) and summer with the lowest number of cases at 3% (16 cases) (Figure 1 and Figure 2).

**FIGURE 3:**
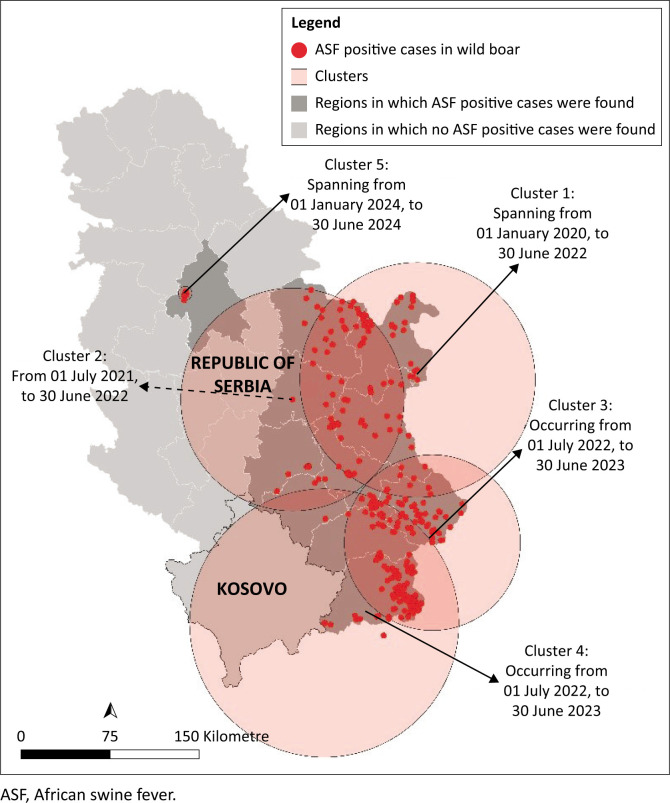
Map of Serbia showing clusters identified through spatiotemporal analysis of African swine fever cases in wild boar. Clusters are highlighted with light red circles and each is labelled with an arrow indicating the cluster name and corresponding time period.

The spatiotemporal analysis identified five distinct clusters (Figure 3):

- *Cluster 1*: Spanning from 01 January 2020 to 30 June 2022, this cluster began with the first recorded case of ASF in wild boar in the eastern part of the country, covering an area of 95 km^2^.
- *Cluster 2*: From 01 July 2021 to 30 June 2022, this cluster was centred in the central region of the country, covering an area of 89 km^2^.
- *Cluster 3*: Occurring from 01 July 2022 to 30 June 2023, this cluster was located in the southeastern region of the country, covering an area of 71 km^2^.
- *Cluster 4*: Occurring from 01 July 2022 to 30 June 2023, this cluster was centred in the southern part of the country near the border with North Macedonia, covering three different regions, with an area of 107 km^2^.
- *Cluster 5*: Spanning from 01 January 2024 to 30 June 2024, this cluster represents a single prolonged outbreak within an enclosed hunting ground, covering an area of 6 km^2^.

**FIGURE 4:**
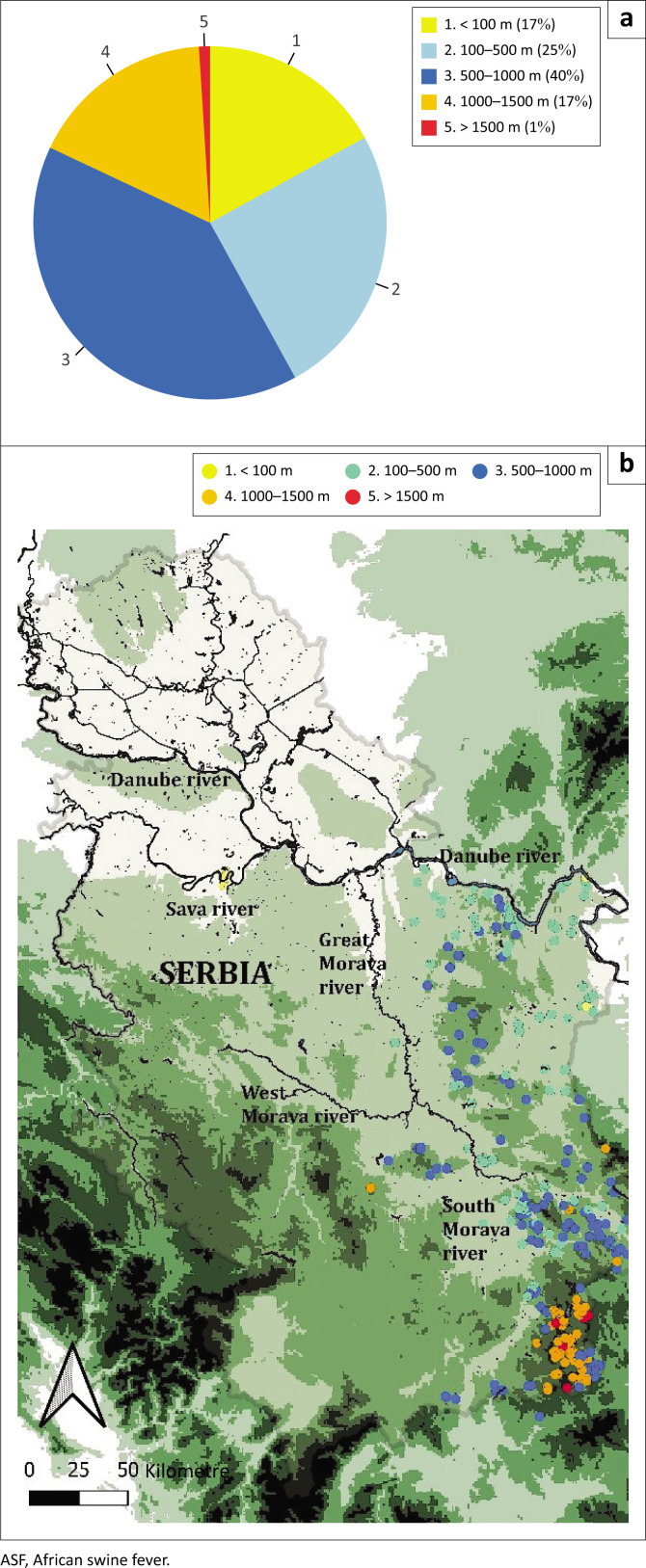
(a) A pie chart showing the percentage distribution of ASF cases across different elevation ranges. (b) A map of Serbia overlaid with an inverted raster elevation file for the country. ASF cases are represented by dots and categorised by the following HTML colour notation: <100 m (yellow, #faff65), 100 m - 500 m (light green, #74e1ab), 500 m - 1000 m (blue, #2175fc), 1000 m - 1500 m (orange, #ff9900), >1500 m (red, #e30035).

Regarding the proximity of ASF outbreaks to roads, 73 (15%) cases were detected within 1 km of a road, 313 (65%) within 5 km and 167 (35%) cases were detected more than 5 km from the nearest road. The Chi-square test yielded a statistical significance (*p* < 0.01), indicating that the distribution of cases is significantly associated with proximity to roads. In terms of proximity to water bodies, 49 (10%) of outbreaks were detected within the nearest vicinity of a water body, 94 (20%) within 1 km and 386 (80%) of outbreaks occurred more than 1 km away from a water body. Chi-square did not yield a statistical significance (*p* > 0.01), suggesting that proximity to water bodies does not significantly influence the distribution of ASF outbreaks. Elevation data analysis revealed the following distribution of ASF outbreaks: 79 (17%) cases were detected in areas below 100 m, 118 (25%) within 100 m – 500 m, 192 (40%) between 500 m and 1000 m, 81 (17%) between 1000 m and 1500 m and 4 (1%) above 1500 m (Figure 4).

## Discussion

Wild boar, native to Europe, are often regarded as ecosystem engineers because of their significant physical impact on habitats. Their feeding behaviour is highly opportunistic, encompassing both plant and animal matter, and varies widely depending on the region in which they are found (Barrios-Garcia & Ballari 2012). Wild boars predominantly inhabit forested regions at elevations between 100 m and 2000 m. The percentage of positive samples of ASF in the affected regions of Serbia has remained steady since the epidemic began in 2020. Despite a significant increase in ASF cases among the domestic pig population, this trend has not been mirrored in the wild boar population. Although ASF is highly lethal in pigs, the persistence of the virus may be because of its relatively low pathogenicity in wild boars and its prolonged environmental survivability. (Podgórski et al. 2020). Similar patterns of low prevalence increases have been reported by other researchers in the Baltic states and Poland (Nurmoja et al. 2017; Podgórski et al. 2020). Regarding seasonality, the majority of cases were detected in winter and spring, consistent with patterns observed in Poland, Slovakia and Hungary (Ståhl et al. 2024). The number of positive samples drops significantly during the summer months, likely because of reduced hunting activity and the rapid decomposition of carcasses, which makes them harder for hunters, foresters and others to detect. Although wild boar hunting is permitted year-round, it primarily focusses on boars and piglets, while hunting sows is banned from March to July. This, combined with extreme summer temperatures, likely contributes to reduced hunting intensity during that period. Additionally, the hunting season for other games typically begins in autumn, which coincides with an increase in the number of submitted samples and detected positive wild boar cases (Srbija šume 2024).

In our spatiotemporal analysis, Clusters 1, 3 and 4 were located near the borders of North Macedonia and Bulgaria, indicating the potential movement of possibly infected wild boar through this region. These clusters are also the largest, extending into significant areas of neighbouring countries. In contrast, Clusters 2 and 5 were centred in the middle of the country, suggesting the potential for the further spread of ASF within Serbia. Notably, Cluster 1 has a very small coverage area of only 6 km^2^, as it represents an enclosed hunting ground. The introduction of ASF into the enclosed hunting ground remains undetermined; however, based on previous studies in the country, it is likely linked to anthropogenic activities (Glišić et al. 2023a, 2023b). In addition, the speed of ASF dissemination has been correlated with wild boar density in the area (Depner et al. 2017). In Serbia, wild boar density decreases from east to west, which may explain why it took 4 years for the disease to be detected beyond the eastern part of the country. To date, no official regulations require hunters or foresters to actively search for wild boar carcasses in hunting grounds or wooded areas. Current government-issued regulations only mandate passive surveillance, where hunters are required to report any carcasses they accidentally come across. Upon notification and confirmation of an ASF-positive carcass, hunting is suspended until further notice. However, this approach relies heavily on the conscientiousness and diligence of hunters in reporting the carcasses they find. As Stončiūtė et al. (2022) found that hunters are often resistant to measures that involve the cessation of hunting activities. Similarly, Rogoll et al. (2023) found that many hunters perceive restriction measures as ineffective. These findings indicate that current strategies may struggle to achieve widespread compliance and effectiveness, potentially hindering efforts to control ASF in wild boar populations. Consequently, the actual number of ASF-positive wild boar is likely significantly higher than the reported cases.

In the Baltic countries during 3 years, over 4000 cases of ASF in wild boar were recorded while in Serbia with a similar wild boar population these numbers are significantly lower. This discrepancy may be attributed to differences in surveillance intensity, wild boar population densities and habitat structures. Notably, the Baltic states have implemented extensive surveillance programmes, which may lead to higher detection rates (Depner et al. 2017). Allepuz et al. (2022) reported that in the EU, wild boar found dead represent the most effective option for ASF detection. However, in our study, the highest number of positive cases was identified in wild boar culled as part of sanitary hunting practices. It is important to notice that the decision on which wild boar to cull is made by hunters, often without standardised criteria. While examining the distribution of wild boar cases in relation to major roads in the country, 65% of the cases were recorded near these roads. This suggests that many more cases likely remain undetected because of the lack of organised search efforts, with most recorded cases being near human settlements and roads. The inadequate surveillance of wooded areas means that the true prevalence of African swine fever virus (ASFV) among wild boars is largely unknown. This oversight can severely hinder disease eradication efforts, as undetected pockets of ASF could persist in forested areas, supported by the high tenacity of the ASFV and the foraging behaviour of wild boar (Chenais et al. 2019; Cukor et al. 2020a). Proximity to waterbodies had no significant impact on the likelihood of detecting wild boar carcasses, a finding consistent with the study by Allepuz et al. (2022). In contrast, Cukor et al. (2020b) reported that the majority of ASF cases were located near water sources. Regarding elevation, the majority of cases were detected at altitudes between 500 m and 1000 m, followed by those between 100 m and 500 m. These elevations align with optimal wild boar habitats characterised by dense forest cover and reduced human activity, which likely facilitates virus persistence and transmission. Although 79 cases were identified below 100 m, these represent instances from a single hunting ground. Such a distribution is expected because the highest wild boar destiny is found in the eastern regions of the country (0.3–0.5) at elevations ranging from 200 m to 2000 m, making it optimal grounds for wild boar (Statistical Office of Serbia 2021).

## Conclusion

In conclusion, the study underscores the persistent challenge of ASF in wild boar populations across Serbia since the epidemic’s onset in 2020. Despite the steady percentage of ASF-positive wild boar, contrasting trends with the significant rise in cases among domestic pigs suggest that the virus persists in wild boar because of its relatively low pathogenicity and prolonged environmental survival. Seasonal patterns reveal a higher incidence of cases during winter and spring, aligning with findings from neighbouring countries. Clusters near borders with North Macedonia and Bulgaria indicate possible cross-border transmission, highlighting the need for collaborative surveillance programmes and harmonised control measures among neighbouring countries. Additionally, the study highlights the critical role of wild boar density and proximity to major roads in the spread of ASF, emphasising the need for more proactive surveillance measures. Current strategies, which rely heavily on voluntary reporting by hunters, may underestimate the true prevalence of the disease. Introducing financial incentives for carcass reporting, mandatory training programmes and integrating geographic information system (GIS)-based tools for real-time surveillance could enhance detection rates. The findings call for enhanced surveillance, particularly in wooded and remote areas, by incorporating drone-based monitoring, GIS mapping of potential hotspots and mandatory carcass reporting. These measures would improve ASF detection and enable targeted control efforts.
